# Health-Related Quality of Life Improvements in Systemic Lupus Erythematosus Derived from a Digital Therapeutic Plus Tele-Health Coaching Intervention: Randomized Controlled Pilot Trial

**DOI:** 10.2196/23868

**Published:** 2020-10-20

**Authors:** Faiz Khan, Nora Granville, Raja Malkani, Yash Chathampally

**Affiliations:** 1 EVP, CityMD Dix Hills, NY United States; 2 Independent Researcher New York, NY United States; 3 Independent Researcher Austin, TX United States; 4 Harris Health Houston, TX United States

**Keywords:** systemic lupus erythematosus, digital health, digital therapeutic, autoimmunity, food as medicine, dietary intervention, health-related quality of life, lifestyle medicine, mobile health, environmental influences on autoimmunity

## Abstract

**Background:**

Systemic lupus erythematosus (SLE), a systemic autoimmune disease with no known cure, remains poorly understood and patients suffer from many gaps in care. Recent work has suggested that dietary and other lifestyle factors play an important role in triggering and propagating SLE in some susceptible individuals. However, the magnitude of influence of these triggers, how to identify pertinent triggers in individual patients, and whether removing these triggers confers clinical benefit is unknown.

**Objective:**

To demonstrate that a digital therapeutic intervention, utilizing a mobile app that allows self-tracking of dietary, environmental, and lifestyle triggers, paired with telehealth coaching, added to usual care, improves quality of life in patients with SLE compared with usual care alone.

**Methods:**

In this randomized controlled pilot study, adults with SLE were assigned to a 16-week digital therapeutic intervention plus usual care or usual care alone. Primary outcome measures were changes from baseline to 16 weeks on 3 validated health-related quality of life (HRQoL) tools: Functional Assessment of Chronic Illness Therapy-Fatigue (FACIT-F), Brief Pain Inventory-Short Form (BPI-SF), and Lupus Quality of Life (LupusQoL).

**Results:**

A total of 50 patients were randomized (23 control, 27 intervention). In per-protocol analysis, the intervention group achieved significantly greater improvement than the control group in 9 of 11 domains: FACIT-F (34% absolute improvement for the intervention group vs –1% for the control group, *P*<.001), BPI-SF-Pain Interference (25% vs 0%, *P*=.02), LupusQoL-Planning (17% vs 0%, *P*=.004), LupusQoL-Pain (13% vs 0%, *P*=.004), LupusQoL-Emotional Health (21% vs 4%, *P*=.02), and LupusQoL-Fatigue (38% vs 13%, *P*<.001) were significant when controlling for multiple comparisons; BPI-SF-Pain Severity (13% vs –6%, *P*=.049), LupusQoL-Physical Health (17% vs 3%, *P*=.049), and LupusQoL-Burden to Others (33% vs 4%, *P*=.04) were significant at an unadjusted 5% significance level.

**Conclusions:**

A digital therapeutic intervention that pairs self-tracking with telehealth coaching to identify and remove dietary, environmental, and lifestyle symptom triggers resulted in statistically significant, clinically meaningful improvements in HRQoL when added to usual care in patients with SLE.

**Trial Registration:**

ClinicalTrials.gov NCT03426384; https://clinicaltrials.gov/ct2/show/NCT03426384

## Introduction

### Background

Systemic lupus erythematosus (SLE) is a multisystem, complex autoimmune disease of unclear etiology affecting at least 1.5 million Americans and 5 million worldwide [1]. The hallmark of the disease is uncontrolled inflammation in otherwise healthy tissue which can lead to organ damage and sometimes even organ failure. SLE can affect any body system—the kidneys, skin, joints, heart, lungs, gastrointestinal system, and nervous system may all become involved, and the pattern of involvement differs from patient to patient. The most common symptoms are fever, rash, profound fatigue, and joint pain and swelling. Disease activity is prone to exacerbations (called flares) alternating with periods of remission in cycles that are often unpredictable and therefore have an even more detrimental effect on quality of life.

There is no cure for SLE and universally effective treatment is not available. Current management relies on immune modulating drugs, but their side effects often increase discomfort and their use carries the risk of severe adverse events [2]. While 5-year survival rates have increased dramatically from 50% to 90% [3,4], patients with SLE still have significantly higher age-standardized mortality rates [5] and lower health-related quality of life (HRQoL) than the general population [6]. Underemployment and work disability, associated primarily with fatigue and pain, are common [7,8]. Young women are disproportionately affected, especially those of non-Caucasian ethnicity and low socioeconomic status. It has recently been reported that SLE is the leading cause of death among chronic inflammatory diseases in women aged 15-24, with death rates exceeding those of HIV and diabetes [9].

Although a clear understanding of the pathophysiology of SLE remains elusive, the recognition that an individual’s DNA blueprint alone does not wholly account for disease occurrence has fueled new areas of research into the environmental and lifestyle determinants of SLE. Important to this line of inquiry is (1) the growing recognition that epigenetic alterations, such as DNA methylation, noncoding RNAs, and histone modifications, are involved in the development of autoimmune diseases [10] and (2) emerging evidence from human and animal studies that these epigenetic processes are influenced by dietary, environmental, and lifestyle factors [11-23]. Modifying these factors presents an attractive, low-risk treatment option for SLE. However, there is much work to be done to better define these potential triggers and determine if eliminating them confers clinical benefit. Complicating these efforts is the fact that SLE is an extremely heterogeneous disease. Widely variable initial presentation, disease course, organ involvement, and response to treatment complicate diagnosis, management, and clinical research efforts. An international team of experts have identified SLE heterogeneity as “the primary barrier hindering advancement” [24]. Given this heterogeneity, it is reasonable to hypothesize that numerous dietary, environmental, and lifestyle SLE triggers exist and differ from patient to patient. Therefore, tracking and analyzing possible trigger–symptom associations require a reliable, accurate, easy-to-use method for gathering and processing a considerable amount of data. Digital therapeutics can accomplish this and may offer unique solutions to the obstacles faced in attempts to address the dietary, environmental, and lifestyle triggers of SLE.

Several digital therapeutics have already received FDA clearance, and many more are in development, to address a range of medical conditions, including prediabetes and diabetes, substance use and opioid use disorders, Alzheimer disease, obesity, hypertension, chronic back pain, attention-deficit/hyperactivity disorder, concussion, and multiple sclerosis [25-27]. Patients with SLE can use several available apps to help track symptoms and disease activity [28-30] and manage medications [31]. However, none of the existing apps have successfully addressed the relationship between dietary, environmental, and lifestyle factors and symptom severity in autoimmunity.

A digital therapeutic platform has been developed which combines self-tracking technology, analytics, and tele-health coaching to identify and remove possible dietary, environmental, or lifestyle triggers, with the goal to provide clinically meaningful improvements in symptoms and HRQoL in those with autoimmune disease. The platform is intended as an adjunct to standard of care.

The platform was developed over several years with extensive feedback from stakeholders in the autoimmune disease community. This has included discussions with patients, family members, physicians, insurance providers, foundations, patient advocacy groups, pharmaceutical companies, and even potential service providers with experience in the sector, such as contract research organizations. The goal has been to commercialize a product that serves an unmet clinical need, but also that fits into the clinical workflow, would be widely adopted, and has a pathway to reimbursement. As a digital therapeutic, the product is also able to track patient usage and engagement during the course of the program, and notifications can be sent following the program to track longer-term outcomes.

Usability and patient preferences have been carefully considered to ensure that a broad range of individuals are comfortable engaging with the smartphone interface, participating in coaching sessions, and complying with suggested interventions throughout the program. Similarly, the web portal and the health coaching protocol itself were iteratively refined through consultation with health coaches and health care providers.

This novel approach is unique in that it implicitly takes disease heterogeneity into account, leverages the growing understanding of the role environment plays in initiating and propagating autoimmune disease, and personalizes each patient’s recommendations based on software data analytics.

### Objective

The objective of this study was to determine whether the addition to usual care of this digital therapeutic program—intended to identify and intervene on dietary and other lifestyle factors found by data analytics to be associated with symptom frequency and severity—improved HRQoL in patients with SLE more than usual care alone.

## Methods

### Study Population

The study enrolled adults (≥18 years) across the United States from December 2017 to May 2018. Participants were recruited through the following online forums: Lupus Friends and Family, Flare Fighter, and Purple Wings Facebook groups; and Clara Health and Autoimmune Registry (online resources for patients interested in participating in clinical trials). Interested individuals completed a prequalification survey online, and only those individuals who passed the prequalification survey (ie, those who were not disqualified) were asked to submit medical records which were reviewed by the study principal investigator (FK) to verify a diagnosis of SLE and confirm all inclusion/exclusion criteria. Eligible individuals underwent a phone consent session and electronically signed an informed consent document if they chose to participate. Participants were assigned to either the intervention or control arm via randomized blocks of 3 to 8 individuals using a cryptographic random seed, targeting 1:1 allocation between groups. After randomization and electronic collection of baseline data, randomization groups were made known to participants.

Inclusion criteria included owning a smartphone, a threshold score for at least one of seven pain and fatigue questions, and taking a stable dose of one of more of the following drugs for 3 or more months prior to study enrollment: immunosuppressive or immunomodulating therapy (biologic or nonbiologic), immunoglobulin therapy, or 20 mg or more of prednisone (or equivalent corticosteroid). Exclusion criteria included pre-existing or incident diagnosis of cancer, pregnancy, or intention to conceive during the study period, and criteria intended to avoid confounding interpretation of changes in outcome: current or planned participation in another interventional or observational study, known plans to alter inclusion criteria medications prior to onset of study or during study, and use of pulse steroids for more than 30 days combined or during the last 4 weeks of the intervention period.

The occurrence of adverse events was assessed during the coaching sessions and by a call to every participant by the principal investigator at the conclusion of the study. Coaches were instructed to convey possible adverse events to a study team physician (FK) who would report them to the Institutional Review Board.

The study was approved by Western Institutional Review Board (CSI: NCT03426384).

### Intervention

**Figure 1:**
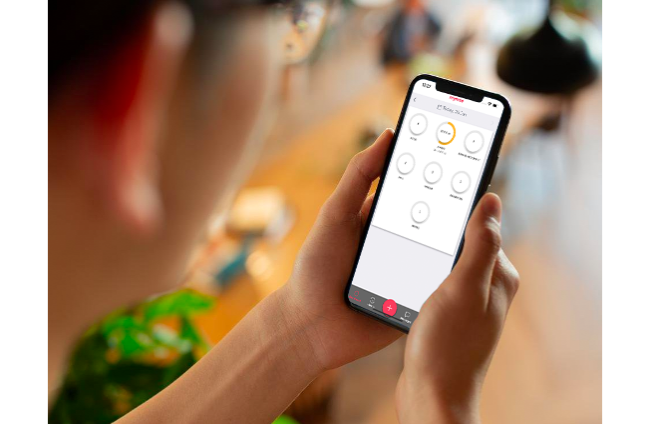
Mobile app for entering symptoms and dietary, environmental, and lifestyle inputs.

The digital therapeutic technology has 3 key components: a smartphone iOS or Android app for the patient to track lifestyle activities (eg, diet, sleep habits, physical activity, bowel movements) and symptoms (Figure 1); software that analyzes and organizes data; and a web portal that presents all patient data to the health coach.

During weekly telehealth coaching sessions, the health coach viewed the data in the web portal and recommended interventions intended to confirm or reject suspected dietary, environmental, and lifestyle triggers. Potential trigger–symptom correlations were presented by the software and the prioritization of triggers and the decision on which intervention to suggest were performed by the health coach. Throughout the program, tracked symptoms were not a comprehensive representation of all participant’s complaints but rather were chosen by the coach based on their propensity to fluctuate, thereby providing indicators which were more likely to be responsive to change. The health coach also provided nutrition and lifestyle education as needed (eg, addition of protein at breakfast, instruction on nutrient-dense foods, recommendations for stress management techniques). Over-the-counter supplementation was recommended by the health coach on a case-by-case basis as appropriate (eg, vitamin D3 if patient records showed deficiency that had not already been addressed—see Multimedia Appendix 1 for full details). A single, certified health coach performed all coaching sessions for every participant. Participants randomized to the intervention group received an email with instructions on how to download the app, create a profile, and use the app to track dietary input. Throughout the study period, participants could receive technology and other support by messaging (through the app) or emailing the study team at any time. After 3-5 days of taking pictures of all the food and beverages consumed daily, participants completed an introductory telephone session to identify their symptoms and goals of the program, review initial tracking data, and receive further training on tracking of other environmental and lifestyle inputs.

Following this initial call, weekly 20-30-minute telehealth coaching sessions were scheduled for the ensuing 15 weeks. Each week, based on associations presented by the software between dietary or other tracked exposures (triggers) and symptoms, the coach suggested behavioral interventions to ameliorate symptoms (eg, eliminate dairy if a patient’s joint pain appeared to flare in relation to dairy intake over the past week). The results of these iterative, weekly interventions were reviewed in subsequent sessions. Compliance with interventions was assessed by analysis of digital tracking and weekly coaching discussions. Successful interventions were maintained, whereas those which did not impact symptoms were either rejected or subjected to longer trials.

Control group participants continued usual care as recommended by their treating physician(s); were not introduced to the intervention app (or any other sham app); and received no training, coaching, or other study interventions.

Prior to entry into the study, all participants had a call with a study staff member to review a summary of the trial, the intervention procedures and schedule, potential risks and benefits, alternative treatments, and provide informed consent. Control participants completed the same battery of assessments at the same intervals as the intervention group participants.  At the end of 16 weeks, control participants met with a study team member by phone during which time final assessment surveys were administered, adverse events over the prior 16 weeks were ascertained, and the opportunity for cross-over to receive the digital therapeutic intervention was offered. All surveys were completed via an HIPAA (Health Insurance Portability and Accountability Act)-compliant version of SurveyGizmo.

### Outcomes

The primary outcomes were changes between baseline and week 16 in 11 domains reflecting various aspects of HRQoL, as assessed by 3 validated patient-reported outcome measures (PROMs): (1) Functional Assessment of Chronic Illness Therapy-Fatigue (FACIT-F), consisting of 13 questions aggregated into 1 domain measuring fatigue; (2) Brief Pain Inventory-Short Form (BPI-SF), consisting of 15 questions classified into 2 domains (pain severity and pain interference), and; (3) Lupus Quality of Life (LupusQoL), consisting of 34 questions classified into 8 domains (fatigue, physical health, planning, burden to others, emotional health, pain, intimate relationships, and body image). All 3 outcome measures have been previously described [32-34] and validated for use in SLE [29,35,36]. The participants were asked to complete these PROMs on a secure website prior to the start of the intervention and at weeks 4, 8, 12, and 16.

Secondary outcomes (derived from analysis of tracking data and coach dashboard information) were tracking adherence (the number of days a participant logged at least one observation into the mobile app in a 24-hour period); session adherence (the number of weekly coaching calls a participant participated in over the 16 weeks); and types and prevalence of (1) the most commonly tracked symptoms, (2) suspected triggers, and (3) interventions. These data were generated from the participant’s tracking data and coaching notes.

### Adherence

Tracking adherence was calculated as the number of days (24-hour period) at least one observation (eg, symptom, food, other lifestyle input) was entered into the app divided by the number of days in the 16-week program (n=112) to arrive at the percentage of days with tracked data (adherence of 100% indicates that the participant used the app to track more than once/day each day of the program). Coaching session adherence was calculated as the number of coaching sessions completed by the participant divided by 16 and converted to a percentage (adherence of 100% indicates that the participant completed 1 or more session/week each week of the program). Median and 25th and 75th percentile values for tracking and session adherence were then calculated for the whole group.

### Statistical Analysis

Prior to the study, the sample size was computed based on the Mann–Whitney *U* test to provide approximately 80% power to detect an effect size proportional to a mean difference in improvement of about 10% with a standard deviation of 10% without correcting for multiple comparisons. This effect size was chosen based on early user experience with the program as well as consideration of previously established minimally important differences for the outcome measures [35-37]. It was determined that a sample size of 50 was sufficient to allow for attrition and still produce the needed power with the remaining participants expected to complete the study. To balance minimizing type I and type II errors, results were highlighted that were significant at an unadjusted significance level of 5% and also, due to the high level of correlation in outcomes, at a level adjusted using the Benjamini–Hochberg method to control the false detection rate at 5% for multiple comparisons.

Nonparametric tests were chosen based on minimal distributional assumptions given the small sample size: Wilcoxon signed-rank test for the change within the intervention and control groups between the baseline and 16-week/end-of-program domain scores; and Mann–Whitney *U* test for changes in score between the intervention and control groups. Medians, 25th, and 75th percentile values are displayed as measures of central tendency and spread. All statistical analyses were performed using IBM SPSS Statistics Subscription (Build 1.0.0.1072).

Intention-to-treat (ITT) analysis included participants who met inclusion criteria at the start of the intervention period, even if they did not complete the study. Per-protocol (PP) analysis was limited to participants who completed 10 or more sessions within the 16-week study period (based on prior exploratory testing), submitted end-of-study data, and experienced no exclusions. Missing follow-up scores from participants who dropped out of the intervention group were populated with the worst observed scores for that time point, thus biasing toward the null hypothesis.

## Results

### Study Population

**Figure 2:**
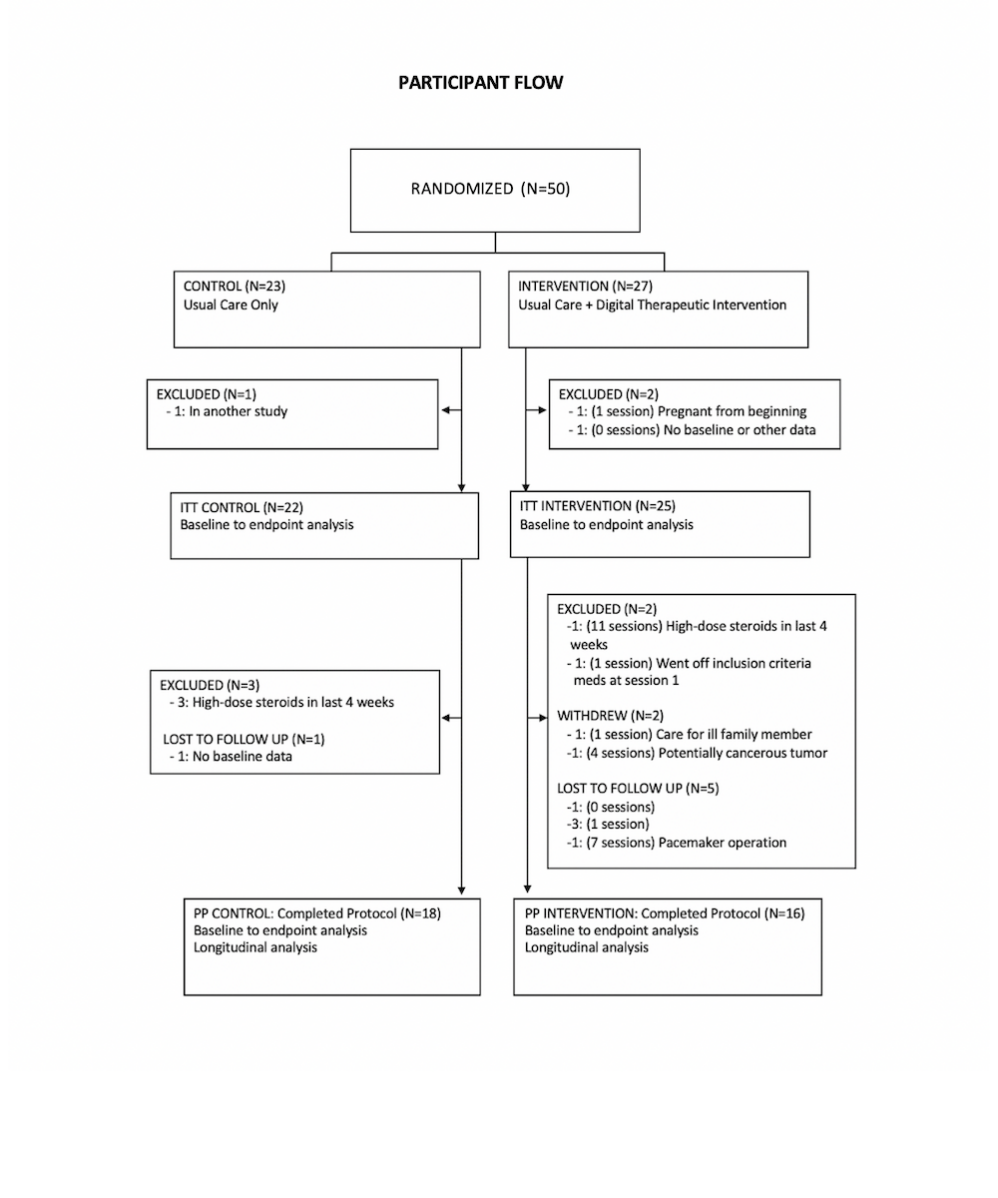
Participant flow. ITT: intention to treat; PP: per protocol.

In total, 50 patients were enrolled, with 47 included in ITT analysis and 34 in PP analysis (Figure 2). Table 1 shows the study population baseline demographics. The control and intervention groups were similar across most categories and any differences were not expected to impact results. For the full cohort, the median age was 43; 96% (44/46) were female; 59% (27/46) of participants were Caucasian, 17% (8/46) Black or African American, and 24% (11/46) Hispanic. Of the 25 ITT intervention participants, 6 (24%) were lost to follow up after completing 0 or 1 coaching sessions (1 discontinued inclusion medication after 1 session; 1 voluntarily withdrew after 1 session to care for a sick family member; 4 were lost to follow up after completing 1 [n=3 participants] or no [n=1] coaching sessions). Of the remaining 19, 16 completed at least ten coaching sessions over 16 weeks (for a completion rate of 84%) and were included in PP analysis. Medications at study entry and baseline scores on the 3 PROMs are shown in Table 2.

**Table 1:** Study population demographics.^a^

Demographics		ITT^b^		PP^c^		EPP^d^			
		Control	Intervention	Control	Intervention	Control	Intervention		
Participants who provided baseline data, N		21	25	18	16	3	9		
Age, median (25th percentile, 75th percentile)		42 (36, 50)	44 (33, 51)	42 (35, 50)	45 (35, 54)	43 (37, 59)	36 (31, 47)		
**Ethnic background**									
	Black or African American	2 (10)	6 (24)	2 (11)	3 (19)	0 (0)	3 (33)		
	Caucasian or White	12 (57)	15 (60)	9 (50)	10 (63)	3 (100)	5 (56)		
	Hispanic or Latino	7 (33)	4 (16)	7 (39)	3 (19)	0 (0)	1 (11)		
**Primary language**									
	English	20 (95)	25 (100)	17 (94)	16 (100)	3 (100)	9 (100)		
	Spanish	1 (5)	0 (0)	1 (6)	0 (0)	0 (0)	0 (0)		
**Gender**									
	Female	20 (95)	24 (96)	17 (94)	15 (94)	3 (100)	9 (100)		
	Male	1 (5)	1 (4)	1 (6)	1 (6)	0 (0)	0 (0)		
**Education level**									
	Some high school	0 (0)	1 (4)	0 (0)	0 (0)	0 (0)	1 (11)		
	High school	1 (5)	0 (0)	1 (6)	0 (0)	0 (0)	0 (0)		
	Some college/trade/technical training	4 (19)	7 (28)	3 (17)	3 (19)	1 (33)	4 (44)		
	Associate degree	5 (24)	6 (24)	4 (22)	5 (31)	1 (33)	1 (11)		
	Bachelor’s degree	9 (43)	5 (20)	8 (44)	3 (19)	1 (33)	2 (22)		
	Master’s/Professional degree	2 (10)	6 (24)	2 (11)	5 (31)	0 (0)	1 (11)		
**Employment**									
	Full-time paid	7 (33)	7 (28)	7 (39)	5 (31)	0 (0)	2 (22)		
	Part-time paid	3 (14)	4 (16)	3 (17)	3 (19)	0 (0)	1 (11)		
	Self-employed	1 (5)	1 (4)	0 (0)	1 (6)	1 (33)	0 (0)		
	Homemaker	1 (5)	0 (0)	1 (6)	0 (0)	0 (0)	0 (0)		
	Out of work, not currently looking	0 (0)	1 (4)	0 (0)	1 (6)	0 (0)	0 (0)		
	Unable to work—on disability	5 (24)	11 (44)	3 (17)	6 (38)	2 (67)	5 (56)		
	Unable to work—other	4 (19)	1 (4)	4 (22)	0 (0)	0 (0)	1 (11)		
**Income Level**									
	US $0-US $25,999	6 (29)	7 (28)	6 (33)	3 (19)	0 (0)	4 (44)		
	US $26,000-US $51,999	5 (24)	7 (28)	3 (17)	5 (31)	2 (67)	2 (22)		
	US $52,000-US $74,999	3 (14)	6 (24)	2 (11)	4 (25)	1 (33)	2 (22)		
	More than US $75,000	7 (33)	5 (20)	7 (39)	4 (25)	0 (0)	1 (11)		
**Relationship status**									
	Life partner (married/other)	13 (62)	15 (60)	11 (61)	11 (69)	2 (67)	4 (44)		
	Single/separate/divorced/widowed	8 (38)	10 (40)	7 (39)	5 (31)	1 (33)	5 (56)		

^a^Values are numbers (percentages) unless stated otherwise.

^b^ITT: intention to treat.

^c^PP: per protocol.

^d^EPP: ITT participants who were excluded from PP.

**Table 2:** Study population inclusion medications and baseline patient-reported outcome measure scores.^a^

Medications and outcome measure scores		ITT^b^		PP^c^		EPP^d^	
		Control	Intervention	Control	Intervention	Control	Intervention
Participants who provided baseline data, N		21	25	18	16	3	9
**Inclusion medications^e^**							
	Azathioprine	2 (10)	5 (20)	1 (6)	1 (6)	1 (33)	4 (44)
	Belimumab	4 (19)	7 (28)	3 (17)	4 (25)	1 (33)	3 (33)
	Hydroxychloroquine	17 (81)	19 (76)	13 (72)	13 (81)	3 (100)	6 (67)
	Immunoglobulin infusions	1 (5)	0 (0)	1 (6)	0 (0)	0 (0)	0 (0)
	Leflunomide	0 (0)	1 (4)	0 (0)	1 (6)	0 (0)	0 (0)
	Methotrexate	4 (19)	3 (12)	4 (22)	2 (13)	0 (0)	1 (11)
	Mycophenolate mofetil	5 (24)	2 (8)	5 (28)	2 (13)	0 (0)	0 (0)
**HRQoL^f^** **, median (25th percentile, 75th percentile)**							
	FACIT^g^-Fatigue	20 (14, 26)	16 (10, 23)	20 (14, 27)	20 (10, 26)	7 (4, 18)	13 (8, 19)
	BPI-SF^h^-Pain Severity	4 (3, 5.7)	5 (3, 6)	3 (3, 5)	4 (3, 6)	5 (5, 7)	6 (3, 7)
	BPI-SF-Pain Interference	5 (5, 6)	6 (4, 7)	5 (4, 6)	6 (4, 7)	7 (3, 7)	6 (6, 8)
	LupusQoL^i^-Physical Health	50 (25, 59)	46 (28, 56)	51 (28, 65)	51 (34, 60)	25 (15, 28)	25 (12, 50)
	LupusQoL-Pain	41 (16, 66)	41 (25, 66)	58 (25, 75)	50 (33, 66)	16 (8, 33)	25 (8, 50)
	LupusQoL-Planning	50 (25, 75)	41 (8, 66)	62 (25, 75)	62 (25, 75)	41 (0, 41)	25 (8, 25)
	LupusQoL-Intimate Relationships	50 (25, 87)	56 (25, 75)	50 (25, 75)	75 (25, 81)	68 (50, 87)	31 (25, 50)
	LupusQoL-Burden to Others	25 (0, 41)	16 (0, 41)	25 (0, 58)	25 (4, 50)	0 (0, 33)	16 (0, 16)
	LupusQoL-Emotional Health	54 (37, 70)	54 (29, 66)	56 (37, 70)	60 (35, 79)	50 (20, 70)	20 (16, 54)
	LupusQoL-Body Image	37 (20, 56)	50 (25, 69)	31 (20, 65)	65 (18, 75)	40 (35, 50)	31 (25, 45)
	LupusQoL-Fatigue	25 (6, 37)	25 (18, 31)	25 (12, 43)	28 (25, 50)	6 (0, 31)	18 (0, 18)

^a^Values are n (%) unless stated otherwise.

^b^ITT: intention to treat.

^c^PP: per protocol.

^d^EPP: ITT participants who were excluded from PP.

^e^Totals do not equal 100% as many patients were on multiple medications.

^f^HRQoL: health-related quality of life.

^g^FACIT-Fatigue: Functional Assessment of Chronic Illness Therapy-Fatigue (FACIT-F); 52-point scale with 0 (worst).

^h^BPI-SF: Brief Pain Inventory-Short Form; 10-point scale with 0 (best).

^i^LupusQoL: Lupus Quality of Life; 100-point scale with 0 (worst).

### Adherence

Table 3 shows tracking and coaching session adherence for the ITT and PP groups. In each group, tracking adherence exceeded 90% and coaching session adherence exceeded 80%. In the ITT group, 16/25 (64%) and 14/25 (56%) participants reached greater than 70% tracking and session adherence, respectively. In the PP group, 16/16 (100%) and 13/16 (81%) participants reached greater than 70% tracking and session adherence, respectively. The percentage of participants achieving 70% or greater tracking and session adherence is reported based on early experience with the platform indicating that this level of engagement correlates with better outcomes.

**Table 3:** Adherence results.

Adherence	Intention to treat			Per protocol	
	Tracking %	Coaching sessions %	Tracking %		Coaching sessions %
Median (25th, 75th percentile)	91.1 (50.9, 97.3)	81.3 (25.0, 81.3)	96.9 (94.4, 99.1)		81.3 (81.3, 93.8)
Over 70% adherence, n/N (%)	16/25 (64)	14/25 (56)	16/16 (100)		13/16 (81)

### Intention-to-Treat Analysis

Within the intervention group, significant improvement over baseline was noted for FACIT-F (median of 26.0 at the end of study vs 16.0 baseline, *P*=.04), LupusQoL-Burden to Others (25.0 vs 16.7, *P*=.02), and LupusQoL-Fatigue (62.5 vs 25.0, *P*=.007). Within the control group, LupusQoL-Burden to Others (41.7 vs 20.8, *P*=.04), LupusQoL-Body Image (45.0 vs 35.0, *P*=.047), and LupusQoL-Fatigue (31.3 vs 25.0, *P*=.03) saw improvement over baseline at 16 weeks. Comparing the 2 groups, although the intervention group improved more than the control group in 6 of 11 domains (FACIT-F, BPI-SF-Pain interference, LupusQoL-Pain, LupusQoL-Emotional Health, LupusQoL-Body Image, and LupusQoL-Fatigue), none of these comparisons reached statistical significance (Table 4). No significant improvements were uncovered when the Benjamini–Hochberg adjustment was applied to the significance level to account for multiple comparisons.

**Table 4:** Intention-to-treat analysis of change in FACIT, BPI-SF, and LupusQoL domain scores from baseline to end of program.^a^

Domain			Within group				Between group		
		Count	Baseline	End of program (EOP)	Change in score (EOP: Baseline)	*P*-value		Difference^b^	*P*-value
**FACIT^c^** **-Fatigue (range 0-52, higher is better)**								4.5	.17
	Intervention	25	16.0 (9.5, 23.5)	26.0 (4.0, 44.0)	4.0 (–3.5, 21.0)	.04^f^			
	Control	22	19.5 (7.0, 26.3)	21.0 (10.5, 28.3)	–0.5 (–5.0, 7.3)	.75			
**BPI-SF^d^** **-Pain Severity (range 0-10, lower is better)**								–0.6	.73
	Intervention	25	5.3 (3.0, 6.8)	5.3 (2.1, 8.3)	0.0 (–2.8, 2.3)	.76			
	Control	22	4.5 (3.0, 6.6)	4.4 (2.6, 7.1)	0.6 (–1.3, 1.0)	.68			
**BPI-SF-Pain Interference (range 0-10, lower is better)**								–0.7	.31
	Intervention	25	6.4 (4.4, 7.9)	4.7 (1.6, 9.3)	–0.6 (–3.6, 0.6)	.16			
	Control	22	5.6 (4.4, 6.7)	5.1 (1.6, 7.5)	0.1 (–1.2, 1.7)	.97			
**LupusQoL^e^** **-Physical Health (range 0-100, higher is better)**								–3.1	.88
	Intervention	25	46.9 (26.6, 56.3)	31.3 (4.7, 78.1)	0.0 (–18.8, 29.7)	.64			
	Control	22	46.9 (23.4, 60.9)	40.6 (21.9, 71.1)	3.1 (–10.2, 10.2)	.82			
**LupusQoL-Pain (range 0-100, higher is better)**								12.5	.21
	Intervention	25	41.7 (20.8, 66.7)	41.7 (0.0, 83.3)	8.3 (–20.8, 33.3)	.63			
	Control	22	37.5 (14.6, 68.8)	33.3 (14.6, 66.7)	–4.2 (–16.7, 2.1)	.28			
**LupusQoL-Planning (range 0-100, higher is better)**								0.0	.24
	Intervention	25	41.7 (8.3, 70.8)	50.0 (0.0, 91.7)	0.0 (–12.5, 25.0)	.38			
	Control	22	45.8 (22.9, 75.0)	41.7 (18.8, 77.1)	0.0 (–27.1, 8.3)	.36			
**LupusQoL-Burden to Others (range 0-100, higher is better)**								–8.3	.92
	Intervention	25	16.7 (0.0, 41.7)	25.0 (0.0, 83.3)	0.0 (0.0, 50.0)	.02^f^			
	Control	22	20.8 (0.0, 45.8)	41.7 (0.0, 77.1)	8.3 (0.0, 16.7)	.04^f^			
**LupusQoL-Intimate Relationships (range 0-100, higher is better)**								–12.5	.46
	Intervention	19	50.0 (25.0, 75.0)	62.5 (0.0, 87.5)	–12.5 (–25.0, 25.0)	.79			
	Control	18	56.3 (25.0, 87.5)	62.5 (25.0, 100.0)	0.0 (–3.1, 12.5)	.47			
**LupusQoL-Emotional Health (range 0-100, higher is better)**								6.2	.37
	Intervention	25	54.2 (25.0, 68.8)	75.0 (4.2, 93.8)	8.3 (–10.4, 29.2)	.30			
	Control	22	52.1 (29.2, 70.8)	56.3 (37.5, 70.8)	2.1 (–12.5, 12.5)	.64			
**LupusQoL-Body Image (range 0-100, higher is better)**								8.1	.505
	Intervention	22	41.3 (23.8, 68.8)	51.9 (0.0, 90.0)	13.1 (–30.3, 21.3)	.76			
	Control	19	35.0 (20.0, 56.3)	45.0 (30.0, 70.0)	5.0 (0.0, 25.0)	.047^f^			
**LupusQoL-Fatigue (range 0-100, higher is better)**								9.4	.22
	Intervention	25	25.0 (15.6, 34.4)	62.5 (0.0, 81.3)	18.8 (–9.4, 43.8)	.007^f^			
	Control	22	25.0 (4.7, 39.1)	31.3 (15.6, 53.1)	9.4 (–6.3, 20.3)	.03^f^			

^a^Within-group values are median (25th percentile, 75th percentile). Nonparametric tests were chosen in order to require minimal distributional assumptions given the small sample size, and medians, 25th, and 75th percentile values are displayed as measures of central tendency and spread in order to be consistent with a nonparametric analysis. The within-group *P*-value is from the Wilcoxon signed-rank test and the between-group *P*-value is from the Mann–Whitney *U* test; *P*-values themselves are unadjusted but the threshold for statistical significance is set using the Benjamini–Hochberg adjustment; LupusQoL-Intimate Relationships and LupusQoL-Body Image allow the possibility of N/A responses.

^b^Difference in median change (intervention – control).

^c^FACIT-Fatigue: Functional Assessment of Chronic Illness Therapy-Fatigue (FACIT-F); 52-point scale with 0 (worst).

^d^BPI-SF: Brief Pain Inventory-Short Form; 10-point scale with 0 (best).

^e^LupusQoL: Lupus Quality of Life; 100-point scale with 0 (worst).

^f^Statistically significant at an unadjusted 2-sided significance level of 5%.

### Per-Protocol Analysis

Within-group analysis of the PP intervention population revealed improvement over baseline at 16 weeks in all domains except LupusQoL-Intimate Relationships at unadjusted significance levels (*P*=.05; Table 5). Adjusting for multiple comparisons, statistically significant improvement was achieved by the PP intervention group in 8 domains: FACIT-F (43.5 at the end of study vs 20.5 baseline; *P*=.001), LupusQoL-Fatigue (81.3 vs 28.1; *P*<.001), LupusQoL-Physical Health (71.9 vs 51.6; *P*=.02), LupusQoL-Planning (83.3 vs 62.5; *P*=.008), LupusQoL-Burden to Others (79.2 vs 25.0; *P*=.003), LupusQoL-Emotional Health (83.3 vs 60.4; *P*=.01), LupusQoL-Body Image (87.5 vs 56.3, *P*=.01), and BPI-SF-Pain Interference (2.0 vs 6.3; *P*=.003). The usual care PP population had significant improvement over baseline for LupusQoL-Fatigue only (34.4 vs 25.0; *P*=.028).

**Figure 3:**
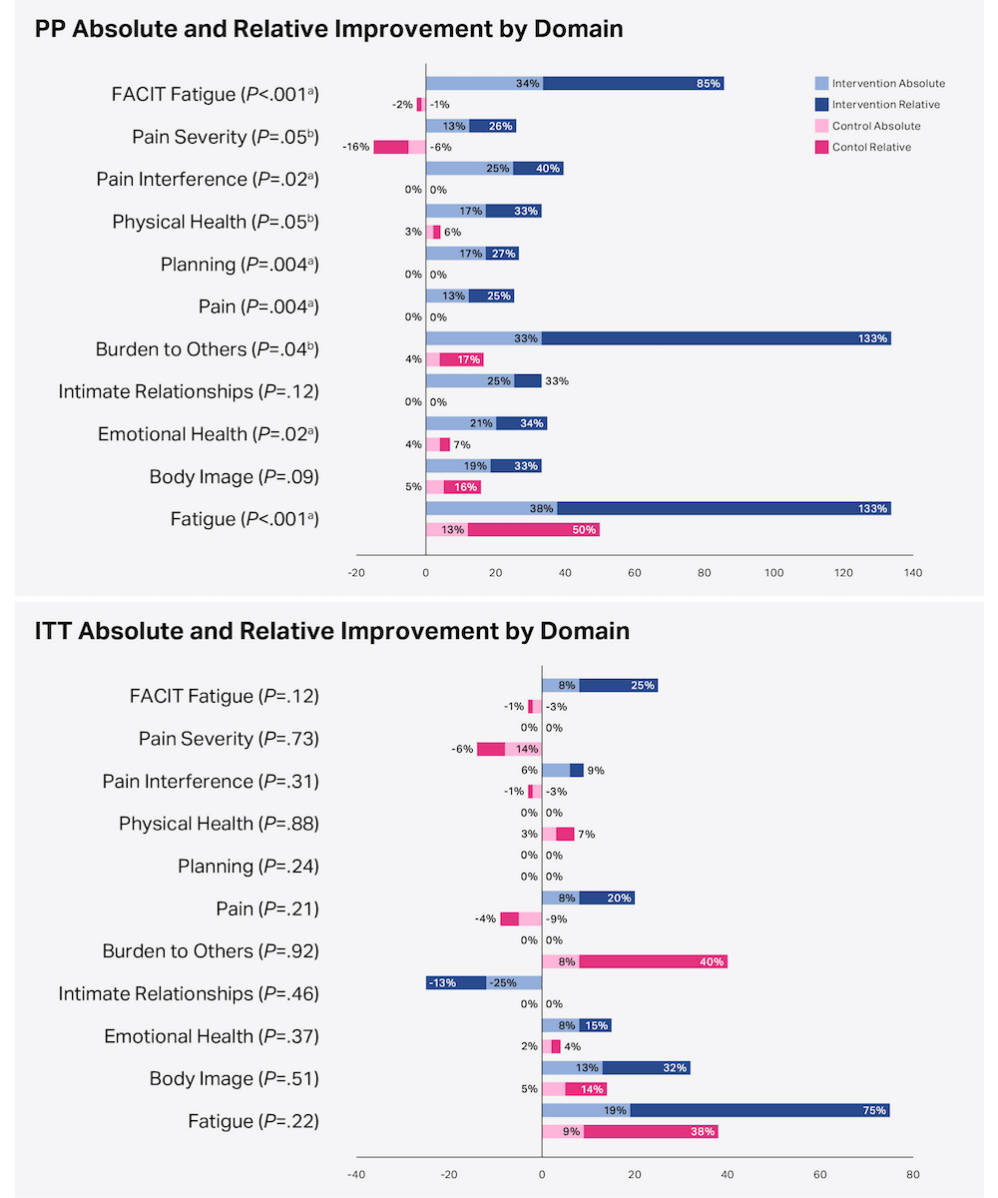
Absolute and Relative Improvement by Domain. Absolute improvement was median change from baseline to endpoint divided by total possible domain score. Relative improvement was median change divided by the median baseline domain score. Changes in BPI-SF-pain interference and BPI-SF-pain severity are converted to positive % for consistency with other domains. *P*-values are from the Mann–Whitney *U* test comparing changes in score between intervention and control groups. *P*-values are unadjusted. Although both ITT intervention and control groups achieved significant improvement in some domains, when the groups were compared, no statistically significant differences were found. ^a^Statistically significant after using the Benjamini–Hochberg adjustment ^b^Statistically significant at an unadjusted two-sided significance level of 5%. FACIT: Functional Assessment of Chronic Illness Therapy; ITT: intention to treat.

Between-group comparisons demonstrated greater improvement in the intervention group than in the control group for every domain. Adjusting for multiple tests, significant differences in favor of the intervention group were reached in 6 domains: FACIT-F (difference in median changes of 18.0, *P*<.001), BPI-SF-Pain interference (–2.5, *P*=.02), LupusQoL-Pain (12.5, *P*=.004), LupusQoL-Planning (16.7, *P*=.004), LupusQoL-Emotional Health (16.7, *P*=.02), and LupusQoL-Fatigue (25.0, *P*<.001). Three additional domains reached significance at an unadjusted level of 5%: BPI-SF-Pain Severity (–1.9, *P*=.049), LupusQoL-Physical Health (14.1, *P*=.049), and LupusQoL-Burden to Others (29.2, *P*=.04). The magnitude of the improvements in all domains (absolute and relative) is shown in Figure 3. Significantly greater improvement was seen in the intervention group compared with the control group. Results on an absolute basis are as follows: FACIT-F (34% intervention vs –1% control, *P*<.001), BPI-SF-Pain severity (13% vs –16%, *P*=.049), BPI-SF-Pain interference (25% vs 0%, *P*=.02), and 4 LupusQoL measures, namely, pain (13% vs 0%, *P*=.004), planning (17% vs 0%, *P*=.004), emotional health (21% vs 4%, *P*=.02), and fatigue (38% vs 13%, *P*<.001).

**Table 5:** Per-protocol analysis of change in FACIT, BPI-SF, and LupusQoL domain scores from baseline to end of program.^a^

Domain			Within group					Between group				
		Count	Baseline	End of program (EOP)	Change in score (EOP – Baseline)	*P*-value		Difference^b^			*P*-value	
**FACIT** ^c^ **-Fatigue (range 0-52, higher is better)**							18.0			<.001^f^		
	Intervention	16	20.5 (10.3, 26.8)	43.5 (28.5, 47.8)	17.5 (4.8, 24.0)	.001^f^						
	Control	18	20.5 (14.0, 27.3)	22.0 (12.5, 28.3)	–0.5 (–4.3, 7.3)	.79						
**BPI-SF^d^ -Pain Severity (range 0-10, lower is better)**							–1.9					.049^g^
	Intervention	16	4.8 (3.0, 6.5)	3.3 (1.6, 5.2)	–1.3 (–3.0, 0.4)	.02^g^						
	Control	18	3.9 (2.9, 5.9)	3.8 (2.6, 6.4)	0.6 (–1.3, 1.0)	.68						
**BPI-SF-Pain Interference (range 0-10, lower is better)**							–2.5					.02^f^
	Intervention	16	6.3 (4.0, 7.5)	2.0 (0.5, 5.3)	–2.5 (–4.4, –0.2)	.003^f^						
	Control	18	5.4 (4.1, 6.3)	4.9 (1.6, 6.5)	0.0 (–1.2, 0.8)	.64						
**LupusQoL^e^ -Physical Health (range 0-100, higher is better)**							14.1		.049^g^			
	Intervention	16	51.6 (34.4, 61.7)	71.9 (37.5, 93.0)	17.2 (0.0, 35.9)	.02^f^						
	Control	18	51.6 (27.3, 65.6)	48.4 (26.6, 71.1)	3.1 (–7.8, 10.2)	.66						
**LupusQoL-Pain (range 0-100, higher is better)**							12.5		.004^f^			
	Intervention	16	50.0 (33.3, 66.7)	83.3 (47.9, 89.6)	12.5 (2.1, 39.6)	.03^g^						
	Control	18	58.3 (22.9, 75.0)	41.7 (16.7, 66.7)	0.0 (–16.7, 2.1)	.23						
**LupusQoL-Planning (range 0-100, higher is better)**							16.7		.004^f^			
	Intervention	16	62.5 (20.8, 75.0)	83.3 (56.3, 100.0)	16.7 (0.0, 41.7)	.008^f^						
	Control	18	62.5 (25.0, 75.0)	41.7 (25.0, 77.1)	0.0 (–27.1, 8.3)	.19						
**LupusQoL-Burden to Others (range 0-100, higher is better)**							29.2		.04^g^			
	Intervention	16	25.0 (2.1, 54.2)	79.2 (31.3, 83.3)	33.3 (0.0, 58.3)	.003^f^						
	Control	18	25.0 (0.0, 60.4)	41.7 (0.0, 77.1)	4.2 (0.0, 16.7)	.11						
**LupusQoL-Intimate Relationships (range 0-100, higher is better)**							25.0		.12			
	Intervention	11	75.0 (25.0, 75.0)	87.5 (75.0, 100.0)	25.0 (–12.5, 50.0)	.06						
	Control	15	62.5 (25.0, 87.5)	50.0 (25.0, 87.5)	0.0 (–12.5, 12.5)	.92						
**LupusQoL-Emotional Health (range 0-100, higher is better)**							16.7		.02^f^			
	Intervention	16	60.4 (34.4, 81.3)	83.3 (68.8, 99.0)	20.8 (4.2, 37.5)	.01^f^						
	Control	18	56.3 (35.4, 71.9)	56.3 (40.6, 67.7)	4.2 (–9.4, 12.5)	.57						
**LupusQoL-Body Image (range 0-100, higher is better)**							13.8		.09			
	Intervention	13	56.3 (14.4, 69.4)	87.5 (68.8, 95.0)	18.8 (13.1, 46.9)	.011^f^						
	Control	15	31.3 (20.0, 65.0)	40.0 (25.0, 70.0)	5.0 (0.0, 23.8)	.12						
**LupusQoL-Fatigue (range 0-100, higher is better)**							25.0		<.001^f^			
	Intervention	16	28.1 (25.0, 53.1)	81.3 (64.1, 92.2)	37.5 (21.9, 48.4)	<.001^f^						
	Control	18	25.0 (10.9, 43.8)	34.4 (25.0, 53.1)	12.5 (–1.6, 20.3)	.03^g^						

^a^Within-group values are median (25th percentile, 75th percentile). Nonparametric tests were chosen in order to require minimal distributional assumptions given the small sample size. Medians, 25th, and 75th percentile values are displayed as measures of central tendency and spread. The within-group *P*-value is from the Wilcoxon signed-rank test; the between-group *P*-value is from the Mann–Whitney *U* test; *P*-values themselves are unadjusted but the threshold for statistical significance is set using the Benjamini–Hochberg adjustment. LupusQoL-Intimate Relationships and LupusQoL-Body Image allow N/A responses.

^b^Difference in median change (intervention – control).

^c^FACIT-Fatigue: Functional Assessment of Chronic Illness Therapy-Fatigue (FACIT-F); 52-point scale with 0 (worst).

^d^BPI-SF: Brief Pain Inventory-Short Form; 10-point scale with 0 (best).

^e^LupusQoL: Lupus Quality of Life; 100-point scale with 0 (worst).

^f^Statistically significant after using the Benjamini–Hochberg adjustment to account for multiple comparisons.

^g^Statistically significant at an unadjusted 2-sided significance level of 5%.

### Per-Protocol Analysis of Longitudinal Change

**Figure 4:**
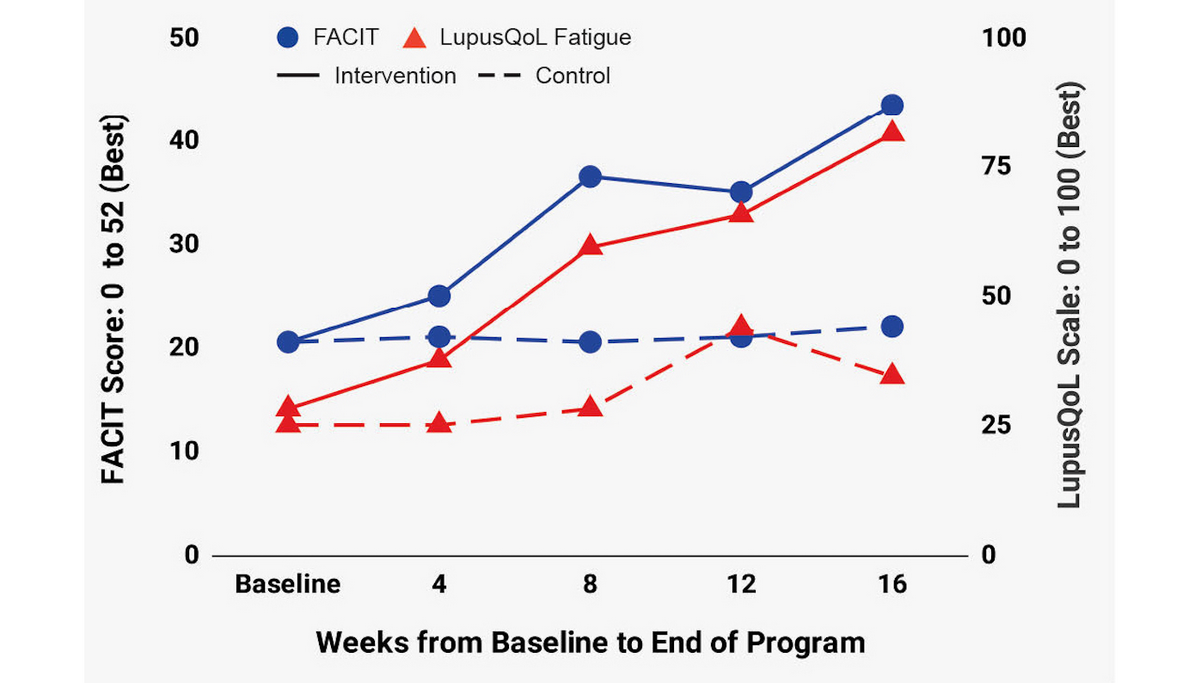
Change over time in FACIT-Fatigue and LupusQoL-Fatigue. FACIT: Functional Assessment of Chronic Illness Therapy; LupusQoL: Lupus Quality of Life.

**Figure 5:**
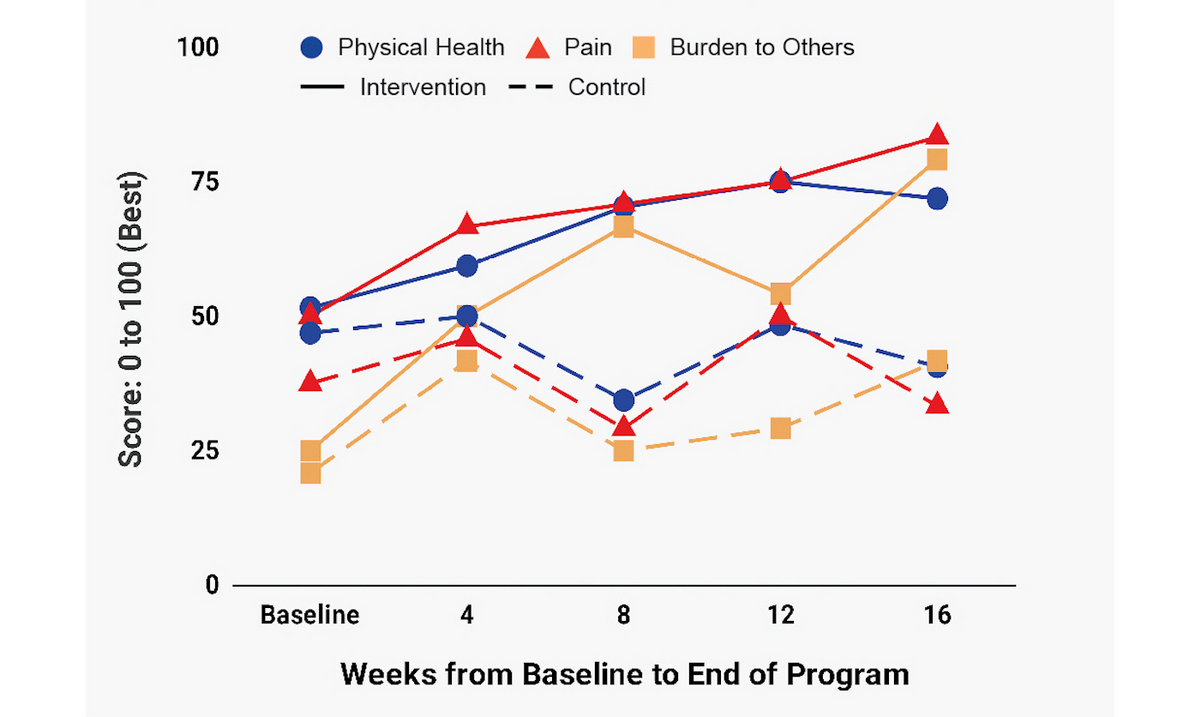
Change over time in LupusQoL-Physical Health, LupusQoL-Pain and LupusQoL-Burden to Others. LupusQoL: Lupus Quality of Life.

**Figure 6:**
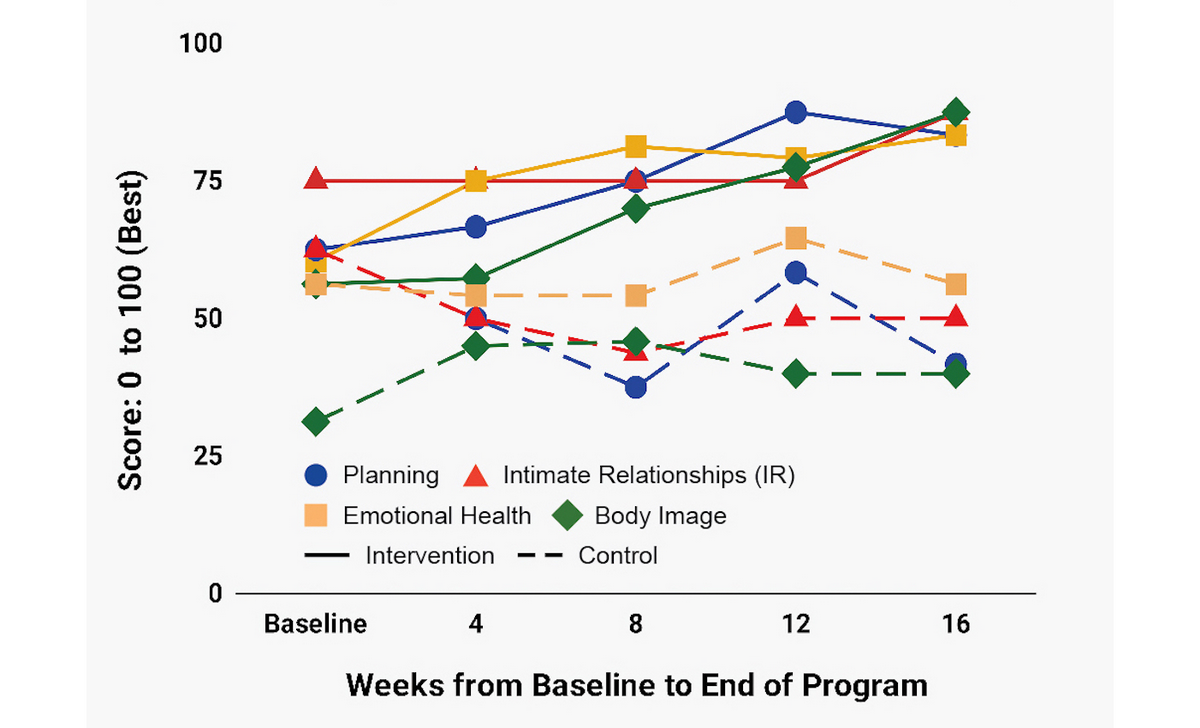
Change over time in LupusQoL-Planning, LupusQoL-Relationships, LupusQoL-Emotional Health and LupusQoL-Body Image. LupusQoL: Lupus Quality of Life.

**Figure 7:**
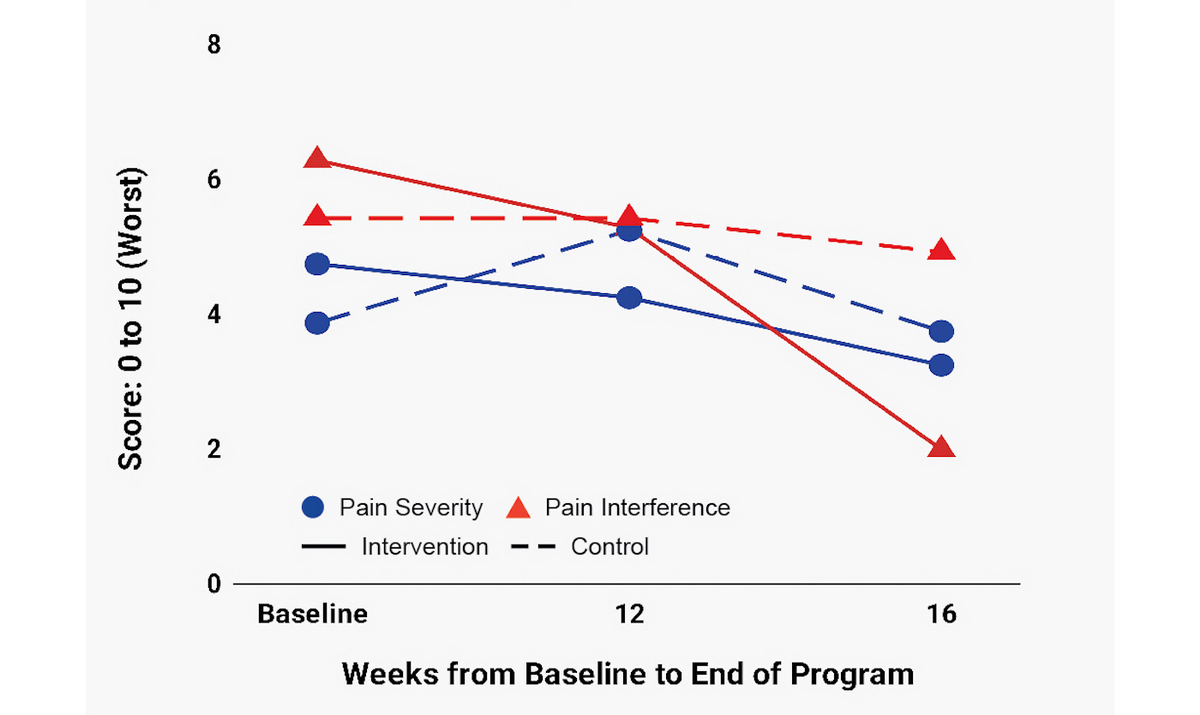
Change over time in BPI-SF Pain Severity and BPI-SF Pain Interference. BPI-SF: Brief Pain Index-Short Form.

Figures 4-7 depict change over time in FACIT-F, LupusQoL, and BPI-SF in both the ITT and PP groups. Improvement in the intervention group started in the first 4 weeks and continued through week 16 with the following exceptions: FACIT-F and LupusQoL-Fatigue had slightly higher improvement rates between weeks 4 and 8 and weeks 12 and 16 (Figure 4); a significant portion of pain reduction occurred between weeks 12 and 16 (Figure 7). The control group experienced modest improvement in LupusQoL-Fatigue at 12 weeks which diminished by week 16, whereas all other domains remained largely unchanged or deteriorated over the course of the 16 weeks.

### Frequency of Tracked Symptoms, Triggers, and Interventions

Table 6 displays the top 3 participant-tracked inputs (symptoms and suspected triggers) and top 4 coach-recommended interventions in the ITT and PP groups. Among both groups, the 3 most frequently tracked symptoms were fatigue, joint pain, and brain fog. The most common triggers in both groups were dairy, gluten, and nightshades (a family of plants that include potatoes, tomatoes, capsicum, bell peppers, eggplant, and tobacco). The most commonly recommended interventions, aside from ensuring adequate hydration, were dietary elimination of triggers and the addition of digestive enzyme supplements, apple cider vinegar, and protein shakes.

**Table 6:** Frequency of tracked symptoms, triggers, and interventions.^a^

Variable			Intention to treat				Per protocol		
			n		Relative frequency (%)		n		Relative frequency (%)
**Interventions**									
	Dietary elimination	17		68		16		100	
	Digestive enzymes	19		76		16		100	
	Protein shake	17		68		15		94	
	Apple cider vinegar	15		60		14		88	
**Triggers**									
	Dairy	19		76		14		88	
	Gluten	14		56		11		69	
	Nightshades	7		28		6		38	
**Symptoms**									
	Joint pain	6		24		6		38	
	Brain fog	6		24		6		38	
	Fatigue	7		28		5		31	

^a^Total population sampled includes intention to treat (N=25) and per protocol (N=16).

### Adverse Events

No adverse events attributable to the intervention occurred. Four participants (3 from the control group and 1 from the intervention group) experienced SLE exacerbations requiring pulse steroids within the last 4 weeks of the study.

## Discussion

### Principal Findings

To the authors’ knowledge, this is the first study to show that a digital therapeutic intervention targeting dietary, environmental, and lifestyle factors can improve HRQoL when added to usual care in patients with SLE. Participants who completed the 16-week protocol showed continuous improvement across all HRQoL domains and statistically greater improvement than those receiving usual care alone for the majority of domains. Of particular interest is the significant improvement noted in fatigue. Fatigue is one of the most debilitating symptoms reported by patients with SLE; is highly correlated with work disability [38], workplace absence (absenteeism), or impaired workplace performance (presenteeism) [39,40]; and is frustratingly recalcitrant to treatment. Although not formally assessed (and therefore only serves as a point for further exploration in future studies), qualitative data collected via coaching notes revealed that 2 participants in the PP intervention group who had been on disability at the start of the study (15 years and 3 years) felt ready for work.

In this SLE population, the most common triggers identified as correlating negatively with symptoms and leading to improvement upon elimination were all dietary—the top 3 being dairy, gluten, and nightshades. While these findings do not provide conclusive evidence linking dairy, gluten, and nightshades to SLE, accumulating data from in vitro, animal, and human studies support the need for ongoing investigation into these potential triggers [41-43].

Elimination of food triggers identified by the program’s software as aggravating symptoms was central to the therapeutic approach of this platform. In addition, a variety of low-risk, nutritional interventions not previously studied in an SLE population were frequently incorporated into the participants’ personalized plans. These interventions were primarily aimed at improving the participants’ numerous digestive and energy-level complaints which weigh heavily on HRQoL in SLE and included digestive enzymes, small amounts of apple cider vinegar, and protein shakes.

Progress in the care of patients with SLE has been slow, largely attributable to inherent disease characteristics as well as health care access and socioeconomic obstacles. As discussed, disease heterogeneity is perhaps the most significant obstacle to progress. Other barriers to advancement are lack of diagnostic, predictive, prognostic, and drug-response biomarkers; ineffective management of SLE due to social determinants of care in predominantly lower socioeconomic status areas; and lack of treatment adherence [24]. Nontraditional solutions to these challenges should be explored and digital therapeutics offer one such novel approach. The digital therapeutic intervention tested here focused on identifying and eliminating dietary, environmental, and lifestyle triggers of SLE as an adjunct to usual care. This approach implicitly takes disease heterogeneity into account, leverages the growing understanding of the role environment plays in initiating and propagating SLE, and personalizes each patient’s recommendations supported by software data analytics. This personalized approach is especially intriguing as it applies to dietary interventions in SLE. A recent review article assessing the significance and impact of dietary factors on SLE pathogenesis found that small and personalized improvements in diet could alter the clinical status of patients with SLE and concluded that “proper diet in SLE can help preserve the body’s homeostasis, increase the period of remission, prevent adverse effects of medication [especially systemic corticotherapy] and improve the patient’s physical and mental well-being” [44].

With on-going research, digital therapeutics may hold the key to overcoming many barriers to SLE care. The enormity of data that can be collected and analyzed via a digital therapeutic platform has the potential to help identify new SLE biomarkers. Aspects of care that prove difficult to deliver to disadvantaged populations with the traditional medical model may be made more accessible. Importantly, if larger studies validate these preliminary findings and build on this work by demonstrating improvements in disease activity measures (eg, Systemic Lupus Erythematosus Disease Activity Index [SLEDAI], SLE Responder Index [SRI]), then dietary and lifestyle interventions delivered in conjunction with a digital therapeutic device may allow for more selective and conservative use of costly, potentially dangerous immune-modulating drugs. Medication changes were not formally followed in this study but information from coaching notes revealed that 5 study participants in the PP intervention group were able to reduce or discontinue immune-modulating medications. In addition, several participants reduced or discontinued use of multiple symptom-relieving medications (including over-the-counter and prescription drugs for gastrointestinal symptoms, pain, depression, and anxiety). As medication usage was not a prespecified outcome in this trial and was not formally assessed, conclusions about the impact of this intervention on medication usage cannot be made. However, if these results are reproduced in larger studies (in which medication information is formally collected and analyzed) the implications of drug reduction alone are important. Polypharmacy is highly prevalent in SLE [45], is associated with elevated risk of adverse drug events, and was shown in a 2017 meta-analysis to be linked to increased mortality [46].

It was not possible in this small pilot trial to examine the underlying physiological mechanisms responsible for patients’ improvements. Provocative findings from several lines of research compel one to consider the effects that the collection of lifestyle modifications, particularly dietary changes, may have had on the health of the intestinal epithelium and the gut microbiome. In animal [14,47-49] and human [50,51] studies, mounting evidence points to a central role of the intestinal epithelial barrier and related diversity and function of the gut microbiome in autoimmune disease. In 2014, the National Institutes of Health (NIH) launched the Integrative Human Microbiome Project “to generate resources to permit comprehensive characterization of the human microbiota to further our understanding of how the microbiome impacts human health and disease” [51]. One of the 3 microbiome-associated conditions which are being explored is autoimmune in nature, namely, inflammatory bowel disease. As microbiome characteristics have also been implicated in SLE, it would be valuable in future studies of this digital therapeutic to assess microbiome composition before and after the intervention.

This exploratory pilot study has many limitations and the results should be interpreted in this context. Physician-scored, validated SLE disease activity measures (such as British Isles Lupus Assessment Group or SLEDAI) were not captured, limiting the capacity to assess disease severity at baseline and change in disease activity by strict clinical criteria throughout the study. In this exploratory pilot study, limited budget and research manpower restricted the ability to pursue this depth of data collection. While inclusion of such clinical disease activity scores in future, larger studies is planned and will provide critical insights, absence of these measures should not diminish the relevance of HRQoL outcomes. The debilitating symptoms, toxicity of immune-modulating treatments, unpredictability of disease activity, and fear of serious, even life-threatening manifestations associated with SLE have a profound impact on HRQoL across multiple domains. These features are not adequately captured by clinical measures of disease activity, which previous research has shown to have poor correlation with patient assessment of HRQoL [52]. Furthermore, HRQoL has been found to be associated with treatment adherence and health care utilization in patients with SLE [53]. The PROMs utilized in this study were chosen to capture many of the diverse domains that contribute to the complex concept of HRQoL.

Selection bias may have been introduced by heavy recruitment from online SLE and other autoimmune patient websites and therefore the study group may not be representative of the general SLE population. However, the number of patients who seek online medical advice is large; continues to grow; and crosses gender, age, and socioeconomic differences [54]. Selection bias may have also been introduced by the requirement of owning a smartphone. Smartphone ownership, however, has become increasingly common across gender, race, education, and economic levels [55], hopefully minimizing this bias. But, in future studies this can be addressed by providing smartphones for those in need.

This study failed to show statistically significant between-group differences in any measured domain in the ITT analysis. These results were affected by disproportionate attrition from the intervention group early in the study (Figure 2). Six intervention participants (6/25, 24%) left the study after having completed 0 to 1 of 16 sessions, whereas only 1 control group participant was lost early. Missing data from participants who dropped out of the intervention group were populated with the worst observed scores for that time point, thus biasing toward the null hypothesis. Furthermore, while 1 participant in the intervention group did receive pulse steroids within the last 4 weeks of the study period, excluding her from PP analysis, 3 patients in the control group also received pulse steroids in this time frame. Any positive effect this treatment had on outcomes would have biased toward the null hypothesis. The attrition rate may speak to the requirements inherent in this type of intervention, namely, that participants need to be motivated and engaged with an aptitude for regular app use and an interest in attending weekly coaching sessions. In future studies, early attrition will be addressed by building a run-in period into the design to help mitigate this issue. That this intervention has shown an 83% completion rate (participating in at least twelve of sixteen coaching sessions) in 70 autoimmune patients from a private insurance cohort is reassuring that the program has acceptable usability (internal data).

As potential adverse events were collected for the control group only at the end of the study (as opposed to the intervention group who were queried about potential adverse events on weekly coaching calls), these data may have been subject to recall bias. Future studies, which are planned to include a sham app and weekly sham coaching calls (see below), will overcome this potential bias.

There was no sham app or sham coaching in this study. Digital apps and health coaching alike are intrinsically engaging, thus vulnerable to the placebo response. It is not possible to tell to what extent HRQoL improvements were influenced by this engagement and patient expectations rather than the program interventions. Future studies should include a convincing sham app and interaction between controls and a health coach at the same frequency as that which occurs with the intervention group. Development of a sham app and sham coaching protocols are underway.

### Conclusions

In conclusion, the digital therapeutic and coaching intervention tested in this pilot trial resulted in statistically significant, clinically meaningful improvements when added to usual care, compared with usual care alone, in several measures of HRQoL (including pain and fatigue) in adult patients with SLE. The study demonstrated that an adaptive, multifaceted intervention which aims at identifying and limiting each SLE participant’s specific dietary and environmental triggers can improve symptoms and HRQoL without additional pharmaceutical manipulation of the immune system. These promising results stimulate a call for a larger study that includes measurement of validated SLE disease activity measures, sham controls, analysis of the biological mechanisms that underlie the improvements, and long-term follow-up of patients to confirm sustained gains in HRQoL. Broad adoption of the intervention could assist in building a database of SLE triggers which could deepen the understanding of the etiology of this disease and potentially contribute to SLE prevention in the future. Finally, given the expected role of diet, environment, and lifestyle in other autoimmune diseases, many of which have gaps in care similar to those in SLE, studies of the intervention’s application to other autoimmune conditions is warranted.

## Appendix

Multimedia Appendix 1 Supplement Protocol.

Multimedia Appendix 2 CONSORT EHEALTH checklist (v 1.6.2).

## Abbreviations

BPI-SF: Brief Pain Index-Short Form
CNS: central nervous system
FACIT-F: Functional Assessment of Chronic Illness Therapy-Fatigue
HIPAA: Health Insurance Portability and Accountability Act
HRQoL: health-related quality of life
ITT: intention to treat
LupusQoL: Lupus Quality of Life
PP: per protocol
PROMs: patient-reported outcome measures
SLE: systemic lupus erythematosus
SLEDAI: Systemic Lupus Erythematosus Disease Activity Index
SRI: SLE Responder Index
