# Supplementary material for: Health-Related Quality of Life Improvements in Systemic Lupus Erythematosus Derived from a Digital Therapeutic Plus Tele-Health Coaching Intervention: Randomized Controlled Pilot Trial
Source: J Med Internet Res. 2020 Oct 20;22(10):e23868. doi: 10.2196/23868 (PMC7609202; doi:10.2196/23868)
Supplement: Multimedia Appendix 1 [file jmir_v22i10e23868_app1.docx]

Health Related Quality of Life Improvements in SLE Derived from a Digital Therapeutic Plus Tele-Health Coaching Intervention: A Randomized Controlled Pilot Trial

Multimedia Appendix 1 – Supplement Protocol

Magnesium

Magnesium supplemented in the following subjects:

1. If subject provides lab work showing magnesium deficiency
2. If subject is having trouble sleeping or is reporting frequent muscle twitches or restless leg syndrome

Depending on a subject’s lifestyle and preferences, magnesium may be taken orally (200mg/day) or used topically via Epsom salt baths (1 daily bath, 2 cups per bath).

Vitamin D

Vitamin D supplemented in the following subjects who are not already on a Vitamin D supplement:

1. If subject provides lab work showing D-vitamin deficiency (25(OH)D <20ng/mL) which was not in the past/currently being repleted, 5000 IU D3 orally daily

Vitamin B12

B12 in methylated form supplemented in the following subjects:

1. If subject provides lab work showing B12 deficiency [<120–180 picomol/L (<170–250 pg/mL)] and/or elevated [methylmalonic acid](https://en.m.wikipedia.org/wiki/Methylmalonic_acid) levels ( >0.4 micromol/L), 5000 mcg orally/daily.

Probiotics

Probiotics supplemented in the following subjects:

1. If subject has recently (within last 6 weeks) been on antibiotics or
2. If subject is experiencing digestive issues

1 capsule per day (Jarro-Dophilus EPS Higher Potency product)

Digestive Enzymes

Digestive enzymes supplemented in the following subjects:

1. If subject has had their gallbladder removed or
2. If subject is experiencing digestive issues or
3. If subject has late night wakening between 1 and 3 am

2 capsules with all meals (Jarro-Zymes product)

Bile Salts

Bile salts supplemented in the following subjects:

1. All subjects without a gallbladder.

2 capsules with all meals (Jarrow Bile Acid Factors product)
