# Supplementary material for: Health-Related Quality of Life Improvements in Systemic Lupus Erythematosus Derived from a Digital Therapeutic Plus Tele-Health Coaching Intervention: Randomized Controlled Pilot Trial
Source: J Med Internet Res. 2020 Oct 20;22(10):e23868. doi: 10.2196/23868 (PMC7609202; doi:10.2196/23868)
Supplement: Multimedia Appendix 2 [file jmir_v22i10e23868_app2.pdf]

CONSORT-EHEALTH Checklist V1.6.2 Report

Manuscript Number: 23868

(based on CONSORT-EHEALTH V1.6), available at [http://tinyurl.com/consort-ehealth-v1-6].

Date completed

9/16/2020 17:43:00

by

Faiz Khan

Health Related Quality of Life Improvements in SLE Derived from a Digital Therapeutic Intervention: A Randomized Controlled Pilot Trial

TITLE

1a-i) Identify the mode of delivery in the title

"A Randomized Controlled Pilot Trial"

1a-ii) Non-web-based components or important co-interventions in title

The Digital Therapeutic Intervention does include a tele-health coaching component which is not included in the title but can be if necessary. New title would be "Health Related Quality of Life Improvements in SLE Derived from a Digital Therapeutic plus Tele-Health Coaching Intervention: A Randomized Controlled Pilot Trial"

1a-iii) Primary condition or target group in the title

"Health Related Quality of Life Improvements in SLE Derived from a Digital Therapeutic Intervention: A Randomized Controlled Pilot Trial"

ABSTRACT

1b-i) Key features/functionalities/components of the intervention and comparator in the METHODS section of the ABSTRACT

"To demonstrate that a digital therapeutic intervention, utilizing a mobile app that allows self-tracking of dietary, environmental, and lifestyle triggers, paired with telehealth coaching, added to usual care, improves quality of life in patients with SLE compared to usual care alone. "

1b-ii) Level of human involvement in the METHODS section of the ABSTRACT

Yes. "To demonstrate that a digital therapeutic intervention, utilizing a mobile app that allows self-tracking of dietary, environmental, and lifestyle triggers, paired with telehealth coaching, added to usual care, improves quality of life in patients with SLE compared to usual care alone. "

1b-iii) Open vs. closed, web-based (self-assessment) vs. face-to-face assessments in the METHODS section of the ABSTRACT

No. Subjects were recruited from multiple sources (online forums, referrals from physicians, referral from friends) making this description cumbersome for abstract.

1b-iv) RESULTS section in abstract must contain use data

"Fifty patients were randomized (23 control, 27 intervention)" Adherence with tracking in app and coaching calls was not included in abstract. It can be added to abstract with the following sentence: "Across all subjects, median adherence with daily tracking in the app exceeded 90% and median adherence with coaching calls exceeded 80%."

1b-v) CONCLUSIONS/DISCUSSION in abstract for negative trials

N/A

INTRODUCTION

2a-i) Problem and the type of system/solution

Yes. "Systemic lupus erythematosus (SLE or lupus) is a multi-system, complex autoimmune disease of unclear etiology affecting at least 1.5 million Americans and 5 million worldwide." "There is no cure for SLE and universally effective treatment is not available." A platform has been developed which combines self-tracking technology, analytics, and telehealth coaching to identify and remove possible dietary, environmental, and/or lifestyle triggers, with the goal to provide clinically meaningful improvements in symptoms and HRQoL in those with autoimmune disease. The platform is intended as an adjunct to standard of care.

2a-ii) Scientific background, rationale: What is known about the (type of) system

No. There is no similar product on the market or that has been tested to our knowledge. However, the basis for looking at diet and environmental factors in SLE is thoroughly discussed ("...emerging evidence from human and animal studies that these epigenetic processes are influenced by dietary, environmental and lifestyle factors.")

Does your paper address CONSORT subitem 2b?

IRB approval not mentioned in Introduction but rather under Methods. " The study was approved by Western Institutional Review Board (CSI: NCT03426384)."

METHODS

3a) CONSORT: Description of trial design (such as parallel, factorial) including allocation ratio

Yes. "After screening and enrollment, patients were assigned to either the intervention or control arm via randomized blocks of 3 to 8 individuals using a cryptographic random seed." There was not mention that this was 1:1 randomization but that can be added if necessary.

3b) CONSORT: Important changes to methods after trial commencement (such as eligibility criteria), with reasons

There were no changes to methods after trial commencement.

3b-i) Bug fixes, Downtimes, Content Changes

4a) CONSORT: Eligibility criteria for participants

Partially.

4a-i) Computer / Internet literacy

Computer/internet literacy were not included in the Inclusion Criteria as subjects only needed to use a mobile app. "Inclusion criteria included owning a smartphone..."

4a-ii) Open vs. closed, web-based vs. face-to-face assessments:

Yes. "Participants were recruited through online lupus and autoimmune forums and direct physician referral." "During weekly telehealth coaching sessions..." "Participants randomized to the intervention group received an email with instructions on how to download and use the app to first track dietary input." "...participants completed an introductory telephone session ..." " weekly 20-30 minute telehealth coaching sessions were scheduled for the ensuing 15 weeks."

4a-iii) Information giving during recruitment

No

4b) CONSORT: Settings and locations where the data were collected

Yes.

4b-i) Report if outcomes were (self-)assessed through online questionnaires

Yes. "The participants were asked to complete these PROMs on a secure website prior to the start of the intervention and at weeks 4, 8, 12, and 16." "All surveys were completed via a HIPAA-compliant version of SurveyGizmo."

4b-ii) Report how institutional affiliations are displayed

There were no institutional affiliations in this study.

5) CONSORT: Describe the interventions for each group with sufficient details to allow replication, including how and when they were actually administered

5-i) Mention names, credential, affiliations of the developers, sponsors, and owners

No authors or evaluators are owners or developers of the software. The standard financial stipend provided to 2 of the authors is disclosed in Conflicts of Interest "Faiz Khan, MD: Standard financial stipends were provided by Mymee, Inc. No other disclosures.
Nora Granville: None declared.
Raja Malkani: Standard financial stipends were provided by Mymee, Inc. No other disclosures.
Yashwant Chathampally, MD: None declared. "

5-ii) Describe the history/development process

Yes. "The platform was developed over several years with extensive feedback from stakeholders in the autoimmune disease community. This has included discussions with patients, family members, physicians, insurance providers, foundations, patient advocacy groups, pharmaceutical companies, and even potential service providers with experience in the sector, such as contract research organizations"

5-iii) Revisions and updating

No.

5-iv) Quality assurance methods

No.

5-v) Ensure replicability by publishing the source code, and/or providing screenshots/screen-capture video, and/or providing flowcharts of the algorithms used

One photo of a screen shot of the subject-facing app is included.

5-vi) Digital preservation

https://apps.apple.com/us/app/mymee-inc/id1261068992

5-vii) Access

Partly. "Participants randomized to the intervention group received an email with instructions on how to download and use the app to first track dietary input." The manuscript does not mention that the app was free to subjects and that they were not paid to participate in the study.

**5-viii) Mode of delivery, features/functionalities/components of the intervention and comparator, and the theoretical framework**		
No.		
**5-ix) Describe use parameters**		
Partially. "...of taking pictures of all the food and beverages consumed daily...". "weekly 20-30 minute telehealth coaching sessions were scheduled for the ensuing 15 weeks"		
**5-x) Clarify the level of human involvement**		
Yes. "...weekly 20-30 minute telehealth coaching sessions were scheduled for the ensuing 15 weeks"		
**5-xi) Report any prompts/reminders used**		
"Following this initial call, weekly 20-30 minute telehealth coaching sessions were scheduled for the ensuing 15 weeks." It is not expolicitly stated that during these coaching calls, the importance of daily app usage was reinforced.		
**5-xii) Describe any co-interventions (incl. training/support)**		
Yes. "Participants randomized to the intervention group received an email with instructions on how to download and use the app to first track dietary input." "After 3-5 days of taking pictures of all the food and beverages consumed daily, participants completed an introductory telephone session to identify their symptoms and goals of the program, review initial tracking data, and provide further training on tracking of other environmental and lifestyle inputs. " "... weekly 20-30 minute telehealth coaching sessions were scheduled for the ensuing 15 weeks."		
**6a) CONSORT: Completely defined pre-specified primary and secondary outcome measures, including how and when they were assessed**		
Partially.		
**6a-i) Online questionnaires: describe if they were validated for online use and apply CHERRIES items to describe how the questionnaires were designed/deployed**		
" All surveys were completed via a HIPAA-compliant version of SurveyGizmo."		
**6a-ii) Describe whether and how "use" (including intensity of use/dosage) was defined/measured/monitored**		
Yes. "Secondary outcomes (derived from analysis of tracking data and coach dashboard information) were tracking adherence (the number of days a participant logged at least one observation into the mobile app in a 24 hour period), session adherence (the number of weekly coaching calls a participant participated in over the 16 weeks) and types and prevalence of: 1) the most commonly tracked symptoms; 2) suspected triggers and; 3) interventions. These data were generated from the participant's tracking data and coaching notes."		
**6a-iii) Describe whether, how, and when qualitative feedback from participants was obtained**		
No		
**6b) CONSORT: Any changes to trial outcomes after the trial commenced, with reasons**		
Yes.		
**7a) CONSORT: How sample size was determined**		
**7a-i) Describe whether and how expected attrition was taken into account when calculating the sample size**		
" ...the sample size was computed based on the Mann-Whitney U test to provide approximately 80% power to detect an effect size proportional to a mean difference in improvement of about 10% with a standard deviation of 10% without correcting for multiple comparisons. This effect size was chosen based on early user experience with the program as well as consideration of previously established minimally important differences for the outcome measures [30,32,]. It was determined that a sample size of 50 was sufficient to allow for attrition and still produce the needed power with the remaining participants expected to complete the study."		
**7b) CONSORT: When applicable, explanation of any interim analyses and stopping guidelines**		
Partially.		
**8a) CONSORT: Method used to generate the random allocation sequence**		
Yes. "...patients were assigned to either the intervention or control arm via randomized blocks of 3 to 8 individuals using a cryptographic random seed."		
**8b) CONSORT: Type of randomisation; details of any restriction (such as blocking and block size)**		
Yes. "...patients were assigned to either the intervention or control arm via randomized blocks of 3 to 8 individuals using a cryptographic random seed."		
**9) CONSORT: Mechanism used to implement the random allocation sequence (such as sequentially numbered containers), describing any steps taken to conceal the sequence until interventions were assigned**		
"...patients were assigned to either the intervention or control arm via randomized blocks of 3 to 8 individuals using a cryptographic random seed."		
**10) CONSORT: Who generated the random allocation sequence, who enrolled participants, and who assigned participants to interventions**		
No		
**11a) CONSORT: Blinding - If done, who was blinded after assignment to interventions (for example, participants, care providers, those assessing outcomes) and how**		
**11a-i) Specify who was blinded, and who wasn't**		
This was not a blinded study		
**11a-ii) Discuss e.g., whether participants knew which intervention was the "intervention of interest" and which one was the "comparator"**		
"Control group participants continued usual care as recommended by their treating physician(s), were not introduced to the intervention app (or any other sham app) and received no training, coaching, or other study interventions."		
**11b) CONSORT: If relevant, description of the similarity of interventions**		
Intervention was compared to treatment as usual.		
**12a) CONSORT: Statistical methods used to compare groups for primary and secondary outcomes**		
Yes. "Missing follow-up scores from participants who dropped out of the intervention group were populated with the worst observed scores for that time point, thus biasing toward the null hypothesis"		
**12a-i) Imputation techniques to deal with attrition / missing values**		
"Missing follow-up scores from participants who dropped out of the intervention group were populated with the worst observed scores for that time point, thus biasing toward the null hypothesis."		
**12b) CONSORT: Methods for additional analyses, such as subgroup analyses and adjusted analyses**		
"Intention to treat (ITT) analysis included participants who met inclusion criteria at the start of the intervention period, even if they did not complete the study. Per protocol (PP) analysis was limited to participants who completed ≥ 10 sessions within the 16-week study period (based on prior exploratory testing), submitted end-of-study data, and experienced no exclusions."		
RESULTS		
**13a) CONSORT: For each group, the numbers of participants who were randomly assigned, received intended treatment, and were analysed for the primary outcome**		
Yes.		
**13b) CONSORT: For each group, losses and exclusions after randomisation, together with reasons**		
"In total, 50 patients were enrolled, with 47 included in ITT analysis and 34 in PP analysis (Figure 2)." "Of the 25 ITT intervention participants, 6 (24%) were lost to follow up after completing 0 or 1 coaching sessions [1 discontinued inclusion medication after 1 session; 1 voluntarily withdrew after 1 session to care for a sick family member; 4 were lost to follow up after completing 1 (3 participants) or no (1) coaching sessions]. Of the remaining 19, 16 completed at least 10 coaching sessions over 16 weeks (for a completion rate of 84%) and were included in PP analysis."		
**13b-i) Attrition diagram**		
Yes. Figure 2 (Participant Flow)		
**14a) CONSORT: Dates defining the periods of recruitment and follow-up**		
Partially.		
**14a-i) Indicate if critical "secular events" fell into the study period**		
No. There were no critical secular events that fell into the study period.		
**14b) CONSORT: Why the trial ended or was stopped (early)**		
N/A.		
**15) CONSORT: A table showing baseline demographic and clinical characteristics for each group**		
Yes.		
**15-i) Report demographics associated with digital divide issues**		
Yes. In Table 1.		
**16a) CONSORT: For each group, number of participants (denominator) included in each analysis and whether the analysis was by original assigned groups**		
**16-i) Report multiple "denominators" and provide definitions**		
Yes. "Table 3 shows tracking and coaching session adherence for the ITT and PP groups. For each participant, tracking adherence was calculated as the number of days (24-hour period) at least 1 observation (e.g., symptom, food, other lifestyle input) was entered into the app, divided by the number of days in the 16-week program (112) to arrive at a percentage of days with tracked data (adherence of 100% indicates that the participant used the app to track ≥ once/day each day of the program). Coaching session adherence was calculated as the number of coaching sessions completed by the participant divided by 16 and converted to a percentage (adherence of 100% indicates that the participant completed ≥1 session/week each week of the program). Median, 25th and 75th percentile values for tracking and session adherence were then calculated for the whole group. In addition, the percent of participants achieving 70% or greater tracking and session adherence are reported based on early experience with the platform indicating that this level of engagement correlates with better outcomes."		
**16-ii) Primary analysis should be intent-to-treat**		

"Within the intervention group, significant improvement over baseline was noted for FACIT-F (median of 26.0 at end of study vs 16.0 baseline, P=0.042), LupusQoL-burden to others (25.0 vs 16.7, P=0.020), and LupusQoL-fatigue (62.5 vs 25.0, P=0.007). Within the control group, LupusQoL-burden to others (41.7 vs 20.8, P=0.038), LupusQoL-body image (45.0 vs 35.0, P=0.047), and LupusQoL-fatigue (31.3 vs 25.0, P=0.029) saw improvement over baseline at 16 weeks. Comparing the 2 groups, although the intervention group improved more than the control group in 6 of 11 domains (FACIT-F, BPI-SF-pain interference, LupusQoL-pain, LupusQoL-emotional health, LupusQoL-body image, and LupusQoL-fatigue), none of these comparisons reached statistical significance (Table 4). No significant improvements were uncovered when the Benjamini-Hochberg adjustment was applied to the significance level to account for multiple comparisons."

17a) CONSORT: For each primary and secondary outcome, results for each group, and the estimated effect size and its precision (such as 95% confidence interval)

Yes. Tables 4 and 5.

17a-i) Presentation of process outcomes such as metrics of use and intensity of use

"Table 3 shows tracking and coaching session adherence for the ITT and PP groups. For each participant, tracking adherence was calculated as the number of days (24-hour period) at least 1 observation (e.g., symptom, food, other lifestyle input) was entered into the app, divided by the number of days in the 16-week program (112) to arrive at a percentage of days with tracked data (adherence of 100% indicates that the participant used the app to track ≥ once/day each day of the program). Coaching session adherence was calculated as the number of coaching sessions completed by the participant divided by 16 and converted to a percentage (adherence of 100% indicates that the participant completed ≥1 session/week each week of the program). Median, 25th and 75th percentile values for tracking and session adherence were then calculated for the whole group. In addition, the percent of participants achieving 70% or greater tracking and session adherence are reported based on early experience with the platform indicating that this level of engagement correlates with better outcomes." And see tables 4 and 5 for clinical outcomes.

17b) CONSORT: For binary outcomes, presentation of both absolute and relative effect sizes is recommended

Yes. Figure 3.

18) CONSORT: Results of any other analyses performed, including subgroup analyses and adjusted analyses, distinguishing pre-specified from exploratory

Yes.

18-i) Subgroup analysis of comparing only users

Yes. All results are presented from ITT and PP groups. See Tables 4 (ITT) and 5(PP). And "Of the 25 ITT intervention participants, 6 (24%) were lost to follow up after completing 0 or 1 coaching sessions [1 discontinued inclusion medication after 1 session; 1 voluntarily withdrew after 1 session to care for a sick family member; 4 were lost to follow up after completing 1 (3 participants) or no (1) coaching sessions]. Of the remaining 19, 16 completed at least 10 coaching sessions over 16 weeks (for a completion rate of 84%) and were included in PP analysis. Medications at study entry and baseline scores on the 3 PROMs are shown in Table 2."

19) CONSORT: All important harms or unintended effects in each group

There were no harms or unintended effects in the groups.

19-i) Include privacy breaches, technical problems

"No adverse events attributable to the intervention occurred. Four participants (3 from the control group and 1 from the intervention group) experienced lupus exacerbations requiring pulse steroids within the last 4 weeks of the study. Of note, the 1 intervention group participant who experienced a flare requiring pulse steroids did not adhere to dietary and lifestyle modifications suggested by the health coach."

19-ii) Include qualitative feedback from participants or observations from staff/researchers

"This exploratory pilot study has many limitations and the results should be interpreted in this context." "Selection bias may have been introduced by heavy recruitment from online lupus and other autoimmune patient websites and therefore the study group may not be representative of the general lupus population. However, the number of patients who seek online medical advice is large, continues to grow and crosses gender, age, and socioeconomic differences [ ]. Selection bias may have also been introduced by the requirement of owning a smartphone. Smartphone ownership has become increasingly common across gender, race, education and economic levels [ ], hopefully minimizing this bias." "The attrition rate may speak to the requirements inherent in this type of intervention – namely, that participants need to be motivated and engaged with an aptitude for regular app use and an interest in attending weekly coaching sessions. In future studies, early attrition will be addressed by building a run-in period into the design to help mitigate this issue. That this intervention has shown an 83% completion rate (participating in at least 12 of 16 coaching sessions) in 70 autoimmune patients from a private insurance cohort is reassuring that the program has acceptable usability (internal data)."

DISCUSSION

20) CONSORT: Trial limitations, addressing sources of potential bias, imprecision, multiplicity of analyses

20-i) Typical limitations in ehealth trials

Yes. "This exploratory pilot study has many limitations and the results should be interpreted in this context." "Selection bias may have been introduced..." "Selection bias may have also been introduced by the requirement of owning a smartphone. " There was no sham app or sham coaching in the present study. Digital apps and health coaching alike are intrinsically engaging thus vulnerable to the placebo response. It is not possible to tell to what extent HRQoL improvements were influenced by this engagement and patient expectations rather than the program interventions."

21) CONSORT: Generalisability (external validity, applicability) of the trial findings

21-i) Generalizability to other populations

"Finally, given the expected role of diet, environment and lifestyle on other autoimmune diseases, many of which have gaps in care similar to those in SLE, studies of the intervention's application to other autoimmune conditions is warranted. "

21-ii) Discuss if there were elements in the RCT that would be different in a routine application setting

There are no significant elements of the RCT that differ from routine use of the application. Interaction with the app, coaches and assessment of outcome measures are similar when app is used routinely.

22) CONSORT: Interpretation consistent with results, balancing benefits and harms, and considering other relevant evidence

22-i) Restate study questions and summarize the answers suggested by the data, starting with primary outcomes and process outcomes (use)

"To the authors' knowledge, this is the first study to show that a digital therapeutic intervention targeting dietary, environmetnal and lifestyle factors can improve HRQoL when added to usual care in patients with SLE. Participants who completed the 16-week protocol showed continuous improvement across all HRQoL domains and statistically greater improvement than those receiving usual care alone for the majority of domains."

22-ii) Highlight unanswered new questions, suggest future research

"While the above findings do not provide conclusive evidence linking dairy, gluten, nightshades or other foods to SLE, accumulating data from in vitro, animal and human studies support the need for ongoing investigation into these potential triggers." "If these results are reproduced in larger studies (in which medication information is more formally collected and analyzed) the implications of drug reduction alone are important." "...it would be valuable in future studies of this digital therapeutic to assess microbiome composition before and after the intervention."

Other information

23) CONSORT: Registration number and name of trial registry

"ClinicalTrials.gov (NCT03426384)"

24) CONSORT: Where the full trial protocol can be accessed, if available

The full trial protocol is not currently publicly accessible.

25) CONSORT: Sources of funding and other support (such as supply of drugs), role of funders

"Funding: This study is supported by funds from the sponsor, Mymee,Inc." "Role of Sponsor :The sponsor provided funding for delivery of the intervention, study management, and stipends made to some study team members; managed the participant recruitment process under PI supervision; and delivered the intervention protocol to intervention participants."

X26-i) Comment on ethics committee approval

No

x26-ii) Outline informed consent procedures

No. The informed consent form can be included in submission if desired.

X26-iii) Safety and security procedures

No

X27-i) State the relation of the study team towards the system being evaluated

"Conflicts of Interest
Faiz Khan, MD: Standard financial stipends were provided by Mymee, Inc. No other disclosures.
Nora Granville: None declared.
Raja Malkani: Standard financial stipends were provided by Mymee, Inc. No other disclosures.
Yashwant Chathampally, MD: None declared."
